# Inflammatory biomarkers predict higher risk of hyperglycemic crises but not outcomes in diabetic patients with COVID-19

**DOI:** 10.3389/fendo.2024.1287795

**Published:** 2024-02-15

**Authors:** Li-Chan Tao, Hong Shu, Ying Wang, Qian Hou, Jian-Jun Li, Xiao-Lin Huang, Fei Hua

**Affiliations:** ^1^ Department of Cardiology, The Third Affiliated Hospital of Soochow University, Changzhou, China; ^2^ Department of Endocrinology, The Third Affiliated Hospital of Soochow University, Changzhou, China; ^3^ State Key Laboratory of Cardiovascular Diseases, Fu Wai Hospital, National Center for Cardiovascular Diseases, Chinese Academy of Medical Sciences and Peking Union Medical College, Beijing, China

**Keywords:** C-reactive protein, procalcitonin, type 2 diabetes mellitus, diabetic ketoacidosis, hyperglycemic hyperosmolar state

## Abstract

**Background:**

Inflammation is a predictor of severe complications in patients with COVID-19 infection under a variety of clinical settings. A few studies suggested that COVID-19 infection was a trigger of hyperglycemic crises including diabetic ketoacidosis (DKA) and/or hyperglycemic hyperosmolar state (HHS). However, the association between inflammation and hyperglycemic crises in diabetic patients with COVID-19 infection is unclear.

**Methods:**

One hundred and twenty-four patients with type 2 diabetes mellitus (T2DM) and COVID-19 infection from January 2023 to March 2023 were retrospectively analyzed. Demographic, clinical, and laboratory data, especially inflammatory markers including white blood cell (WBC), neutrophils, neutrophil-to-lymphocyte ratio (NLR), c-reactive protein (CRP) and procalcitonin (PCT) were collected and compared between patients with or without DKA and/or HHS. Multivariable logistic regression analysis was conducted to explore the association between inflammatory biomarkers and the prevalence of hyperglycemic crises. Patients were followed up 6 months for outcomes.

**Results:**

Among 124 diabetic patients with COVID-19, 9 were diagnosed with DKA or HHS. Comparing COVID-19 without acute diabetic complications (ADC), patients with DKA or HHS showed elevated levels of c-reactive protein (CRP, *P*=0.0312) and procalcitonin (PCT, *P*=0.0270). The power of CRP and PCT to discriminate DKA or HHS with the area under the receiver operating characteristics curve (AUROC) were 0.723 and 0.794, respectively. Multivariate logistic regression indicated 1.95-fold and 1.97-fold increased risk of DKA or HHS with 1-unit increment of CRP and PCT, respectively. However, neither CRP nor PCT could predict poor outcomes in diabetic patients with COVID-19.

**Conclusion:**

In this small sample size study, we firstly found that elevated serum CRP and PCT levels increased the risk of hyperglycemic crises in T2DM patients with COVID-19 infection. More study is needed to confirm our findings.

## Introduction

There exhibited a biphasic association between diabetes mellitus (DM) and severe acute respiratory syndrome coronavirus 2 [SARS-CoV-2, coronavirus disease 2019 (COVID-19)], especially type 2 diabetes mellitus (T2DM). On the one hand, DM has been identified as a crucial risk factor for increased rate of hospital admission, developing severe pneumonia, and substantial mortality compared to patients without DM (1, 2), although the underlying pathophysiology is not fully understood. On the other hand, current studies so far indicate that SARS-CoV-2 may contribute to metabolic dysfunction and impaired glucose homeostasis (3, 4), including acute metabolic complications such as diabetic ketoacidosis (DKA) (4, 5) and hyperglycemic hyperosmolar state (HHS) (6) in diabetic patients.

Although recent studies suggest that COVID-19 can trigger or accelerate diabetes-related hyperglycemic emergencies (7, 8), evidence regarding parameters affecting the occurrence of DKA and/or HHS in diabetic patients with COVID-19 is scarce. Attempting to find the culprit, we currently acknowledge that DKA is characteristically more common in type 1 diabetes mellitus (T1DM), although it can occur in patients with T2DM with specific triggers such as severe infection (9) or treatment of sodium-glucose cotransporter-2 (SGLT-2) inhibitors (10). HHS is a life-threatening endocrine emergency that most commonly affects patients with T2DM. Infection is also identified as a well- documented precipitating factor for HHS in established diabetes (11). Hence, the aim of the study was to explore the association of inflammatory biomarkers with the prevalence of DKA or HHS in T2DM patients with COVID-19 and their clinical outcomes, contributing to provide the theory basis for the early diagnosis and therapeutic intervention of hyperglycemic crises.

## Research design and methods

### Study participants

This is a retrospective cohort study involving T2DM patients who were hospitalized with COVID-19 in the Third Affiliated Hospital of Soochow University from January 2023 to March 2023. The inclusion criteria were: (1) diagnosed with T2DM according to the criteria of Chinese Diabetes Society (12) and World Health Organization (13); (2) diagnosed with COVID-19 by a positive nasopharyngeal test of SARS-CoV-2. The exclusion criteria were: (1) patients aged < 18 years old; (2) patients with pregnancy; (3) patients with major mental illness. Before the start of the study, all the participants signed written informed consent forms. The study was approved by the Institutional the Ethics Committee and Review Committee of the Third Affiliated Hospital of Soochow University.

### Definitions

Definition of DKA: (1) plasma glucose > 13.9 mmol/L; (2) with positive serum or urine ketones; (3) arterial pH < 7.3, serum bicarbonate < 18mmol/L and anion gap (AG) >10mmol/L. The diagnosis of DKA was confirmed according to the American Diabetes Association (ADA) consensus guidelines (14).

Definition of HHS (14): (1) plasma glucose >33.3 mmol/L; (2) arterial pH > 7.3, serum bicarbonate > 18mmol/L, AG < 12mmol/L, strongly positive urine glucose, negative or weakly positive urine ketone, normal or slightly higher blood ketone; (3) effective plasma osmolality > 320 mOsm/kg; (4) complicated with conscious disturbance, delirium or epileptiform convulsion.

### Clinical and biochemical measurements

Baseline clinical characteristics including sex, age, weight, smoking or drinking status, significant comorbidities including asthma, chronic obstructive pulmonary disease (COPD), hypertension, cardiovascular disease, and others, other detailed medical history were also recorded on the day of admission.

After fasting for 10 hours, peripheral venous blood was extracted before administration of corresponding treatment on the morning after admission. Briefly, serum procalcitonin (PCT), c-reactive protein (CRP), D-dimer, aspartate aminotransferase (AST), alanine aminotransferase (ALT), creatinine (Cr), blood urea nitrogen (BUN), total cholesterol (TC), triglyceride (TG), low-density lipoprotein cholesterol (LDL-C), high-density lipoprotein cholesterol (HDL-C), and fasting blood glucose (FBG) were analyzed by an automatic analyzer.

### Follow-up and outcomes assessment

Patients were followed up 6 months after discharged from hospital through telephone interviews. Medical record information system was used to confirm health outcomes further. Participants were tracked from baseline to date of death or readmission or Aug 2023, whichever occurred first.

### Statistical analysis

Data were organized and analyzed using SAS version 9.3 (SAS Institute Inc, Cary, NC). First, continuous variables were evaluated by using the Kolmogorov-Smirnov test to ensure whether they conformed to a normal distribution. Continuous variables that followed a normal distribution were presented as mean ± standard deviation (SD), whereas data that did not conform to normal contribution were presented as medians ± interquartile ranges. Categorical variables were illustrated as proportions. Parameters did not conform to normal contribution were first processed with log transformation. Comparisons between two groups were analyzed by independent sample T-Test. Differences between proportions were analyzed by the Chi-square test. Receiver operating characteristic (ROC) analysis was conducted to determine the diagnostic power of inflammatory biomarkers for the presence of DKA or HHS. Linear regression was conducted to identify the link between inflammatory indicators and potential confounding factors for ADC.

To explore the association between inflammatory parameters and the risk of ADC and clinical outcomes among diabetic patients with COVID-19, multivariable logistic regression was performed. First, for the inflammatory indicators vary with sex and age, Model 1 was adjusted for sex and age. Previous studies suggested that lifestyle was a contributing factor for worse glycemic control and diabetes-related hyperglycemic emergencies (15), thus model 2 was adjusted for smoking and drinking status based on Model 1. Owing to the effects of metabolic status, liver and renal function on the development of DKA or HHS (16–19), model 3 was adjusted for the status of hypertension, BMI, LDL-C, HDL-C, ALT, AST, and creatinine on the basis of Mode 2. Finally, considering that use of antibiotics could have a huge impact on the outcomes of patients with severe infection, model 4 was adjusted for the use of antibiotics or not on the basis of Model 3.

All the Tests were two-sided and *P*-value < 0.05 was considered as significant.

## Results

### Characteristics of study population according to status of hyperglycemic crises

This study included 124 T2DM patients who were admitted to hospital with COVID-19 infection. The mean age of total populations was 73.6 ± 9.7 years old, and the proportion of males was 60.48% (n=75). Of these, 9 of 124 (7.26%) diabetic patients were diagnosed with DKA or HHS. **Table 1** showed the clinical characteristics of the study populations based on the status of hyperglycemic crises. Comparing COVID-19 without acute diabetic complications, patients with DKA or HHS showed metabolic disturbance and hypercoagulable state, as indicated by elevated levels of TG, TC, LDL-C and D-dimer (P<0.05 for each). It is worth noting that increased levels of inflammatory parameters including PCT and CRP were significantly more common among patients with COVID-19 and DKA or HHS.

**Table 1:** Characteristics of study population according to status of acute diabetic complications.

Variables	Without acute diabetic complications (N=115)	With acute diabetic complications (N=9)	*P* value
Age (years)	74.0 ± 9.4	68.7 ± 12.8	0.1149
Males [N (%)]	70 (60.8)	5 (55.6)	0.74
Current smoker [N (%)]	7 (6.09)	2 (0.13)	0.13
Current drinker [N (%)]	4 (3.48)	1 (11.11)	0.32
BMI (kg/m^2^)	24.09 ± 3.06	23.31 ± 5.70	0.6273
Diabetes duration (years)	10 (6-20)	7 (1.88-25)	0.567
Blood biochemical parameters			
TG (mmol/L)	1.07 (0.87-1.48)	1.79 (1.34-3.41)	<.0001
TC (mmol/L)	3.98 ± 1.01	5.16 ± 1.96	0.0037
LDL-C (mmol/L)	2.40 ± 0.72	3.03 ± 1.45	0.0313
HDL-C (mmol/L)	1.07 ± 0.29	1.04 ± 0.42	0.8150
Blood glucose at admittance (mmol/L)	12.22 ± 5.63	26.66 ± 11.75	0.0061
FPG (mmol/L)	8.86 (6.45-12.98)	6.95 (5.38-12.20)	0.2879
ALT (U/L)	20.0 (14.3-33.8)	17.0 (12.3-22.4)	0.5542
AST (U/L)	24.3 (17.6-32.7)	22.3 (17.6-30.8)	0.7601
Serum creatinine (μmol/L)	84.10 ± 43.14	84.27 ± 42.96	0.99
Hemoglobin (g/l)	123.49 ± 17.26	139.22 ± 19.61	0.0102
Hematocrit (%)	36.38 ± 4.93	41.77 ± 5.42	0.0021
WBC (*10^9^/L)	6.48 ± 2.59	8.36 ± 4.32	0.0504
Neutrophils (*10^9^/L)	4.89 ± 2.44	6.69 ± 4.35	0.0483
NLR	4.14 (2.77-6.67)	7.32 (4.57-10.45)	0.65
PCT (ng/mL)	0.13 (0.07-0.25)	0.50 (0.27-0.86)	0.0270
CRP (mg/L)	24.25 (5.75-66.90)	91.40 (39.10-135.90)	0.0312
D-dimer (mg/L)	0.63 (0.35-1.12)	1.16 (0.52-2.74)	0.0243
Types of comorbidities N (%)			
Asthma	1 (0.9)	0 (0)	1.0000
COPD	1 (0.9)	0 (0)	1.0000
Hypertension	86 (74.8)	7 (77.8)	1.0000
Heart disease	28 (24.3)	1 (11.1)	0.6209
Others	62 (53.9)	6 (66.7)	0.6946
**Antibiotics use**	106 (92.2)	7 (77.8)	0.144
Past treatment strategies N (%)			
SGLT2 inhibitors	3 (2.6)	0 (0)	1.0000
ACEI/ARB/ARNI	29 (25.2)	1 (11.1)	0.6862
Anticoagulant	1 (11.1)	0 (0)	1.0000
Antiplatelet	19 (16.5)	3 (33.3)	0.4131
Statin	13 (11.3)	2 (22.2)	0.6624

Data were presented as mean ± SD for median (interquartile ranges) for continuous variables, and numbers (proportions) for categorical variables.

*P* values were calculated by t test for continuous variables and Chi-square test or Fisher's precision probability test for categorical variables.

BMI, body mass index; TG, triglyceride; TC, total cholesterol; LDL-C, low-density lipoprotein cholesterol; HDL-C, high-density lipoprotein cholesterol; FBG, fasting blood glucose; ALT, alanine aminotransferase; AST, aspartate aminotransferase; eGFR, estimated glomerular filtration rate; WBC, white blood cell; NLR, neutrophil-to-lymphocyte ratio; PCT, procalcitonin; CRP, c-reactive protein; COPD, Chronic obstructive pulmonary disease; SGLT2 inhibitors, sodium-glucose cotransporter 2 inhibitors; ACEI/ARB/ARNI, angiotensin-converting enzyme inhibitors/Angiotensin II receptor blockers/ angiotensin receptor II blocker-neurolysin inhibitor.

### Inflammatory indicators discriminate the presence of DKA or HHS

**Figure 1:**
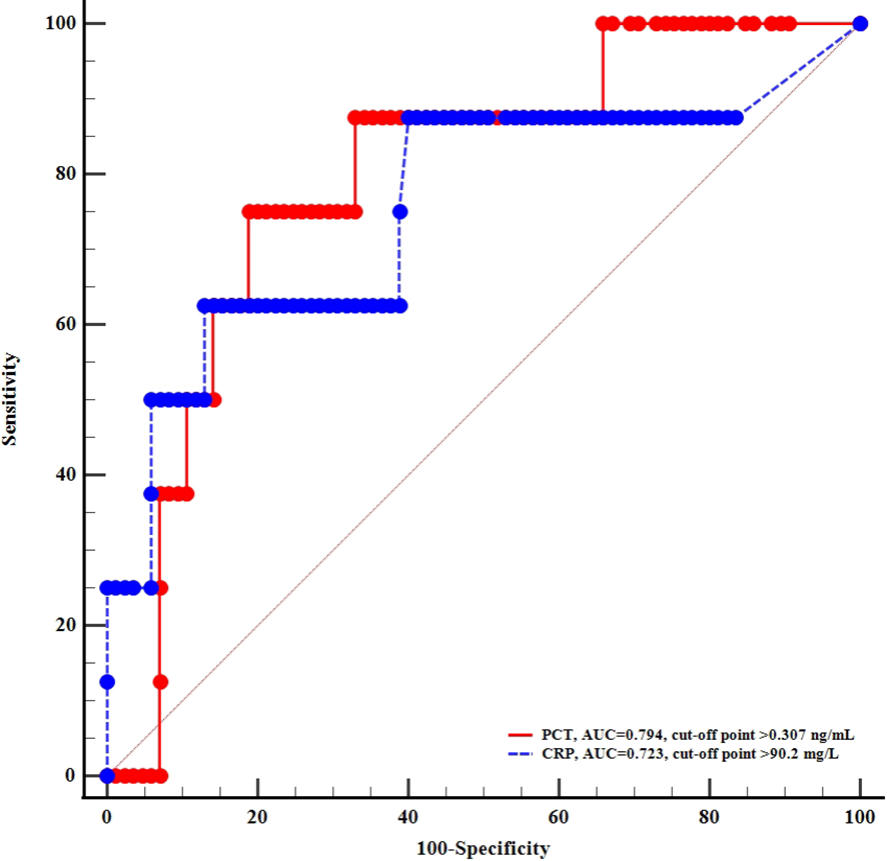
ROC curves of c-reactive protein and procalcitonin for diagnosing acute diabetic complications.

To explore the association between inflammation and hyperglycemic crises during the infection of COVID-19, we performed ROC analysis and found that CRP and PCT exhibited high power of discrimination for DKA or HHS, with the area under curve (AUC) of 0.723 and 0.794, respectively (**Figure 1**).

### Inflammatory biomarkers and the risk of hyperglycemic crises

**Figure 2:**
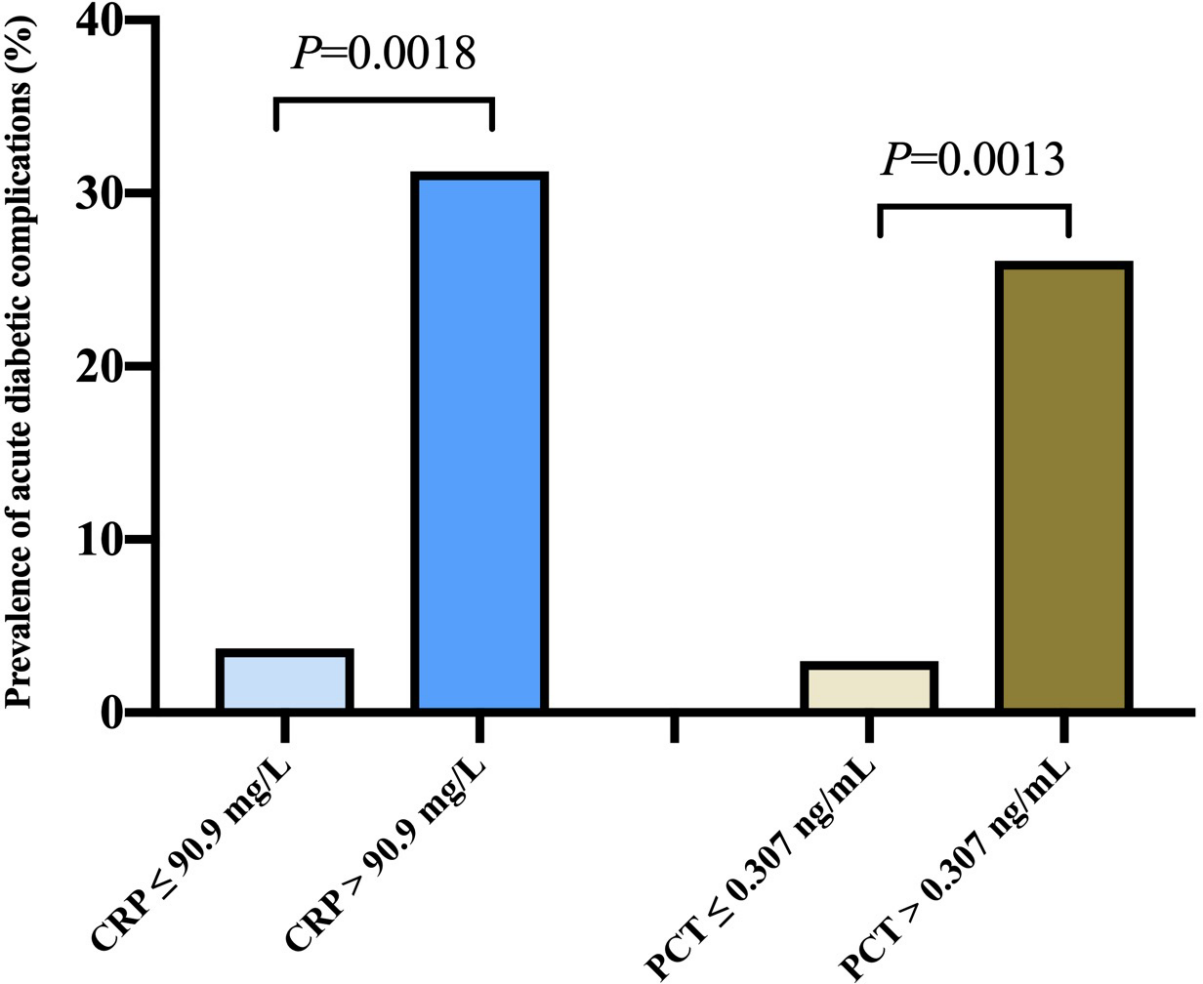
Prevalence of acute diabetic complications according to different levels of c-reactive protein and procalcitonin levels.

**Figure 2** exhibited the prevalence of hyperglycemic crises based on different levels of CRP and PCT. According to the cut-off points of CRP in ROC analysis, the prevalence of DKA or HHS in diabetics with COVID-19 were 3.7% *vs* 31.25% (P<0.05). Likewise, the prevalence of DKA or HHS tended to increase with the elevation of PCT (2.97% *vs* 26.09%, P< 0.05).

As demonstrated in **Table 1**, in addition to inflammatory indicators, metabolic disturbance and hypercoagulable state were exhibited in diabetics with acute complications during COVID-19. To identify the association of CRP, PCT and these potential confounding factors, linear regression analysis was conducted and found that both CRP and PCT were positively associated with log-transformed D-dimer (**Table 2**).

**Table 2:** Associations of CRP and PCT levels with metabolic and coagulation characteristics.

Variables mmol/L or mg/L	Log-transformed CRP		Log-transformed PCT	
	*β* ± SE	*P*	*β* ± SE	*P*
Log-transformed FPG (mmol/L)	0.040 ± 0.047	0.40	0.014 ± 0.041	0.73
TyG index	0.010 ± 0.061	0.87	0.021 ± 0.055	0.71
Log-transformed TG (mmol/L)	-0.041 ± 0.042	0.34	0.003 ± 0.039	0.93
TC (mmol/L)	-0.130 ± 0.079	0.11	0.040 ± 0.073	0.59
LDL-C (mmol/L)	-0.140 ± 0.067	0.032	0.013 ± 0.063	0.83
HDL-C (mmol/L)	0.015 ± 0.026	0.58	0.028 ± 0.024	0.24
Log-transformed D-dimer (mg/L)	0.270 ± 0.089	0.0033	0.280 ± 0.083	0.0012

*P* values calculated using generalized linear regression, adjusting age, sex, smoking and drinking status, BMI, history of hypertension, TG, HDL-C, log-transformed ALT, serum creatinine and log-transformed D-dimer.

CRP, c-reactive protein; PCT, procalcitonin; FBG, fasting blood glucose; TyG, triglyceride-glucose; TG, triglyceride; TC, total cholesterol; LDL-C, low-density lipoprotein cholesterol; HDL-C, high-density lipoprotein cholesterol.

Then multivariate logistic regression was conducted to further assess the associations between CRP, PCT and the prevalence of hyperglycemic crises in T2DM patients with COVID-19 infection. **Table 3** showed that in age- and sex-adjusted model, the risk of DKA or HHS increased with 1-unit increment of CRP, as well as PCT. Likewise, after adjusting for the status of smoking and drinking habits, BMI, history of hypertension, LDL-C, HDL-C, AST, and serum creatinine, 1.95-fold, 1.57-fold increased risk of DKA or HHS were exhibited with 1-unit increment of CRP and PCT, respectively. Finally, after adjusting for antibiotics use or not, the risk of DKA or HHS increased with 2.27-fold increasement of CRP and 1.67-fold increasement of PCT.

**Table 3:** Odds ratios of acute diabetic complications according to inflammation related indicators.

	Each 1-unit increase in inflammation related indicators [OR (95% CI)]	
	Log-transformed CRP	Log-transformed PCT
Model 1	2.49 (1.17-5.30)	1.96 (1.12-3.43)
Model 2	2.79 (1.21-6.42)	2.32 (1.22-4.43)
Model 3	2.95 (1.12-7.79)	2.57 (1.06-6.22)
Model 4	3.27 (1.12-9.52)	2.67 (1.09-6.51)

Data were presented as OR (95% CI) calculated by multivariable logistic regression.

Model 1: Adjusted for age and sex;

Model 2: Further adjusted for smoking and drinking status on basis of model 1;

Model 3: Further adjusted for BMI, history of hypertension, LDL-C, HDL-C, log-transformed ALT and serum creatinine on basis of model 2;

Model 4: Further adjusted for using antibiotics or not on basis of model 3.

CRP, c-reactive protein; PCT, procalcitonin; BMI, body mass index; LDL-C, low-density lipoprotein cholesterol; HDL-C, high-density lipoprotein cholesterol; ALT, alanine aminotransferase.

### Predictive performance of inflammatory indicators for clinical outcomes

**Tables 4**, **5** showed the predictive performance of CRP and PCT for clinical outcomes in patients followed up 6 months. However, neither CRP nor PCT could predict readmission or death in diabetic patients with COVID-19 infection.

**Table 4:** Baseline inflammation related indicators of participants according to status of readmission or death.

Variables	Non-readmission/death (N=79)	Readmission/death (N=45)	*P* value
PCT (ng/mL)	0.14 (0.08-0.26)	0.11 (0.04-0.22)	0.72
CRP (mg/L)	24.8 (5.00-73.90)	17.2 (6.90-58.20)	0.91

Data were presented as mean ± SD for median (interquartile ranges) for continuous variables, and numbers (proportions) for categorical variables.

*P* values were calculated by t test for continuous variables and Chi-square test or Fisher's precision probability test for categorical variables.

WBC, white blood cell; NLR, neutrophil-to-lymphocyte ratio; PCT, procalcitonin; CRP, c-reactive protein.

**Table 5:** Odds ratios of readmission or death according to baseline inflammation related indicators of participants.

	Each 1-unit increase in inflammation related indicators [OR (95% CI)]	
	Log-transformed CRP	Log-transformed PCT
Model 1	0.96 (0.66-1.39)	0.92 (0.68-1.24)
Model 2	0.95 (0.65-1.39)	0.92 (0.68-1.24)
Model 3	1.04 (0.66-1.66)	1.06 (0.70-1.60)

Data were presented as OR (95% CI) calculated by multivariable logistic regression.

Model 1: Adjusted for age and sex;

Model 2: Further adjusted for smoking and drinking status on basis of model 1;

Model 3: Further adjusted for BMI, history of hypertension, LDL-C, HDL-C, log-transformed ALT and serum creatinine on basis of model 2.

CRP, c-reactive protein; PCT, procalcitonin; BMI, body mass index; LDL-C, low-density lipoprotein cholesterol; HDL-C, high-density lipoprotein cholesterol; ALT, alanine aminotransferase.

## Discussion

In the retrospective single-center study, we identified risk factors linked with hyperglycemic crises including DKA and HHS in patients with COVID-19. Inflammatory biomarkers CRP levels greater than 90.9 mg/L, and PCT levels greater than 0.307 ng/mL were significantly associated with the prevalence of diabetes-related hyperglycemic emergencies. However, neither CRP nor PCT was associated with poor outcomes in diabetic patients with COVID-19.

In our cohort, including 124 diabetic patients with COVID-19, 9 of 124 (7.26%) diabetic patients were diagnosed with acute diabetic complications including DKA or HHS. To the best of our knowledge, there is currently no previously reported study in literature exploring risk factors associated with the occurrence of diabetes-related metabolic emergencies in this cohort of patients. Previous studies have suggested a high incidence of DKA in patients hospitalized with severe COVID-19 infection. Of these, in a retrospective study of 218 patients with COVID-19 in the UK, results showed that an overall prevalence of DKA was 2% (4 cases), while the prevalence increased to 7% among patients with DM (20). However, this review enrolled patients with both T1DM and T2DM and had no separate group of HHS. Among current reported case series of COVID-19 infection with DKA and/or HHS, most patients were diagnosed with T1DM while few had confirmed T2DM with COVID-19 infection (8, 21, 22). We have purposefully excluded T1DM patients and other DM isoforms including new onset DM after COVID-19 to ensure homogeneity of our populations.

During the process of infection and inflammation, CRP and PCT are remarkable biomarkers. CRP, a protein originated from the liver, was suggested to be elevated in diabetic patients compared to non-diabetic patients during the infection of COVID-19. CRP accounted for 32.7% of the total link between DM and poor COVID-19 prognosis, including severe pneumonia status admitting to Intensive Care Unit (ICU) and longer inpatients stay (23). On the other hand, study by Mondal found that serum CRP level was elevated in COVID-19 patients with DKA, similar to our findings (24). PCT, has been suggested to be increased in severe and FETAL COVID-19, as well as in COVID-19 patients with new-onset DKA (24). In our study, elevated CRP and PCT levels were associated with higher risk of DKA or HHS in T2DM with COVID-19 infection, confirming that a hyper-inflammatory response might mediate the link between diabetes and COVID-19. Diabetes is known as a chronic inflammatory response, persistent hyperglycemia would incite a proinflammatory and prooxidative status which are associated with acute and chronic complications (25). In diabetic patients, an exaggerated immune reaction following COVID-19 infection, coupled with a preexisting heightened inflammatory states (25), may partly explain the association between inflammatory indicators and ADC. However, Mondal et al. suggested that there was a significant incidence of new-onset DKA with parenteral glucocorticoids therapy (potent anti-inflammatory drugs) in T2DM patients with COVID-19 (24). Thus, analysis evaluating the treatment of glucocorticoids in pre-existing acute diabetic complications is warranted to further explore the association between inflammatory and acute diabetic complications in COVID-19. Furthermore, owing to impaired innate and cell-mediated immune response in DM, diabetic patients are likely to suffer an increased susceptibility to additional bacterial infections (26). However, even if combined with bacterial infections (after adjusting for use of antibiotics), CRP and PCT were significantly associated with the prevalence of DKA or HHS. Therefore, in addition to a preexisting proinflammatory response and superimposed hospital-acquired infections, COVID-19 patients with higher levels of CRP and PCT may be preferred to maintain sharp vigilance for the occurrence of diabetes-related hyperglycemic emergencies.

Various studies showed that elevated CRP levels in COVID-19 patients might indicate excessive inflammatory stress contributing to severe/critical illness or even death (27–29). PCT is also indicated as a sensitive biomarker in predicting COVID-19 progression and mortality (30, 31). However, in this study, no positive prognostic values of CRP and PCT were detected in diabetic patients with COVID-19 infection for 6 months follow-up. This inconsistence might be attributed to different recruited subjects, diverse comorbidities of enrolled population, and the potential impacts of drugs. Furthermore, current studies regarding the predictive performance of CRP and PCT were focused on short-term prognosis of COVID-19 infection (27), whereas the association between inflammatory biomarkers with long-term prognosis in diabetic patients with COVID-19, especially with ADC remains uncertain. Therefore, more large sample studies in the future may improve the reliability of inflammatory markers in predicting the prognosis of patients with diabetes and COVID-19 infection.

Even though our study identified interesting findings, we do respect the limitations. First, this is a single center study with a relatively small sample size, thus the function of confounders predicting severe events of DM might be underestimated. Besides, heterogeneity as well as selection bias might be unavailable. Furthermore, the present study confirms the vital role of inflammatory response in acute diabetic complications during the infection of COVID-19, prospective studies with larger number are warranted to evaluate the potential therapeutic effects of antibiotic or anti-inflammatory treatment in diabetics with COVID-19 infection.

## Conclusions

This study consistently demonstrates that inflammatory indicators CRP and PCT are associated with the prevalence of hyperglycemic crises in T2DM patients with COVID-19, confirming the significant role of inflammation in the pathogenesis of ADC in COVID-19. Further explorations are urgent to understand other mechanisms which contribute to the hyperglycemic crises observed in patients with COVID-19 and the underlying associations with inflammation.

## Data availability statement

The original contributions presented in the study are included in the article/supplementary material. Further inquiries can be directed to the corresponding author.

## Ethics statement

The studies involving humans were approved by The Third Affiliated Hospital of Soochow University. The studies were conducted in accordance with the local legislation and institutional requirements. The participants provided their written informed consent to participate in this study.

## Author contributions

L-CT: Formal analysis, Funding acquisition, Writing – original draft. HS: Investigation, Methodology, Writing – original draft. YW: Data curation, Writing – original draft. FH: Supervision, Writing – review & editing. X-LH: Formal analysis, Supervision, Validation, Writing – review & editing. J-JL: Writing – review & editing. QH: Data curation, Methodology, Writing – original draft.
